# D-Ribose Induces Cellular Protein Glycation and Impairs Mouse Spatial Cognition

**DOI:** 10.1371/journal.pone.0024623

**Published:** 2011-09-08

**Authors:** Chanshuai Han, Yang Lu, Yan Wei, Ying Liu, Rongqiao He

**Affiliations:** 1 State Key Laboratory of Brain and Cognitive Sciences, Institute of Biophysics, Chinese Academy of Sciences, Beijing, China; 2 Key Laboratory of Mental Health, Institute of Psychology, Chinese Academy of Sciences, Beijing, China; 3 Graduate University of the Chinese Academy of Sciences, Beijing, China; 4 School of Life Sciences, University of Science and Technology of China, Anhui, China; Emory University, United States of America

## Abstract

**Background:**

D-Ribose, an important reducing monosaccharide, is highly active in the glycation of proteins, and results in the rapid production of advanced glycation end products (AGEs) *in vitro*. However, whether D-ribose participates in glycation and leads to production of AGEs *in vivo* still requires investigation.

**Methodology/Principal Findings:**

Here we treated cultured cells and mice with D-ribose and D-glucose to compare ribosylation and glucosylation for production of AGEs. Treatment with D-ribose decreased cell viability and induced more AGE accumulation in cells. C57BL/6J mice intraperitoneally injected with D-ribose for 30 days showed high blood levels of glycated proteins and AGEs. Administration of high doses D-ribose also accelerated AGE formation in the mouse brain and induced impairment of spatial learning and memory ability according to the performance in Morris water maze test.

**Conclusions/Significance:**

These data demonstrate that D-ribose but not D-glucose reacts rapidly with proteins and produces significant amounts of AGEs in both cultured cells and the mouse brain, leading to accumulation of AGEs which may impair mouse spatial cognition.

## Introduction

Non-enzymatic glycation of proteins by reducing saccharides such as D-glucose (Glc) and D-ribose (Rib) is a post-translational modification process [1], leading to the formation of fructosamine [2] and advanced glycation end products (AGEs) [3]. The role of Glc in the glycation of proteins has been widely studied, however the role of other reducing monosaccharides such as Rib in glycation and their resulting effects on cell metabolism has received much less attention.

D-ribose is a naturally occurring pentose monosaccharide present in all living cells including the blood and is a key component of many important biomolecules such as riboflavin (i.e., vitamin B2) [4], ribonucleic acid (RNA) [5], and adenosine tri-phosphate (ATP) [6]. As a reducing monosaccharide, Rib has the ability to react with proteins to produce glycated derivatives. Glycation with Rib (ribosylation) gives rise to AGEs more rapidly than glycation with Glc which requires a relatively long time [7]. Rib, however, is also closely associated with many fundamental processes in cellular metabolism. For this reason, glycation of proteins with Rib needs to be addressed and investigated.

The rate of glycation depends upon monosaccharide concentration and anomerization rate and is inversely proportional to the number of carbon atoms in the reducing monosaccharide [8]. Under physiological conditions, the anomerization rate of Rib is much higher than that of Glc. The aldofuranose five-membered ring of Rib is not planar but occurs in one of a variety of conformations generally described as “puckered” [9]. This unstable aldofuranose ring is vulnerable to reactions with amino groups, giving rise to its high efficiency in protein glycation. Therefore, comparing ribosylation with glucosylation should provide new clues for clarifying some of the important complications caused by advanced glycation end products in vivo.

*In vitro* studies on the role of Rib in glycation have been carried out. Rib can glycate rat tail tendon collagen *in vitro* and the structure of the collagen is significantly altered by Rib-induced glycation [10]. Luciano and colleagues prepared glycated fetal calf serum with Rib and found that while ribosylation reduces the proliferation of pancreatic islet beta-cells, cell necrosis and cell apoptosis rate increase correspondingly [11]. Ribosylated bovine serum albumin polymerizes and forms globule-like aggregates with high cytotoxicity [12]. However, the relationship between ribosylation and neurodegenerative diseases is still unknown.

In this laboratory we have observed that glycation induces inactivation and conformational change of D-glyceraldehyde-3-phosphate dehydrogenase [13], [14]. We have also compared the characteristics of ribosylation on neuronal Tau protein [15], and α-synuclein [16] with those of glucosylation *in vitro*, showing that ribosylation occurs much more rapidly than glucosylation. Ribosylated neuronal protein is much more cytotoxic than glucosylated protein. Nevertheless, little is known about whether Rib can rapidly induce glycation in cells and the AGEs produced by ribosylation can impair the cognitive function. Here, we treated cultured cells and mice with Rib and Glc to compare ribosylation and glucosylation for the production of AGEs. We found that Rib reacted rapidly with proteins and produced significant amounts of AGEs in cultured cells and mouse brain tissues, and that accumulation of AGEs impaired mouse spatial cognition. This finding implies that Rib-derived AGEs may be related to impairments of learning and memory ability.

## Results

### D-ribose decreases cell viability and leads to high yields of AGEs

**Figure 1:**
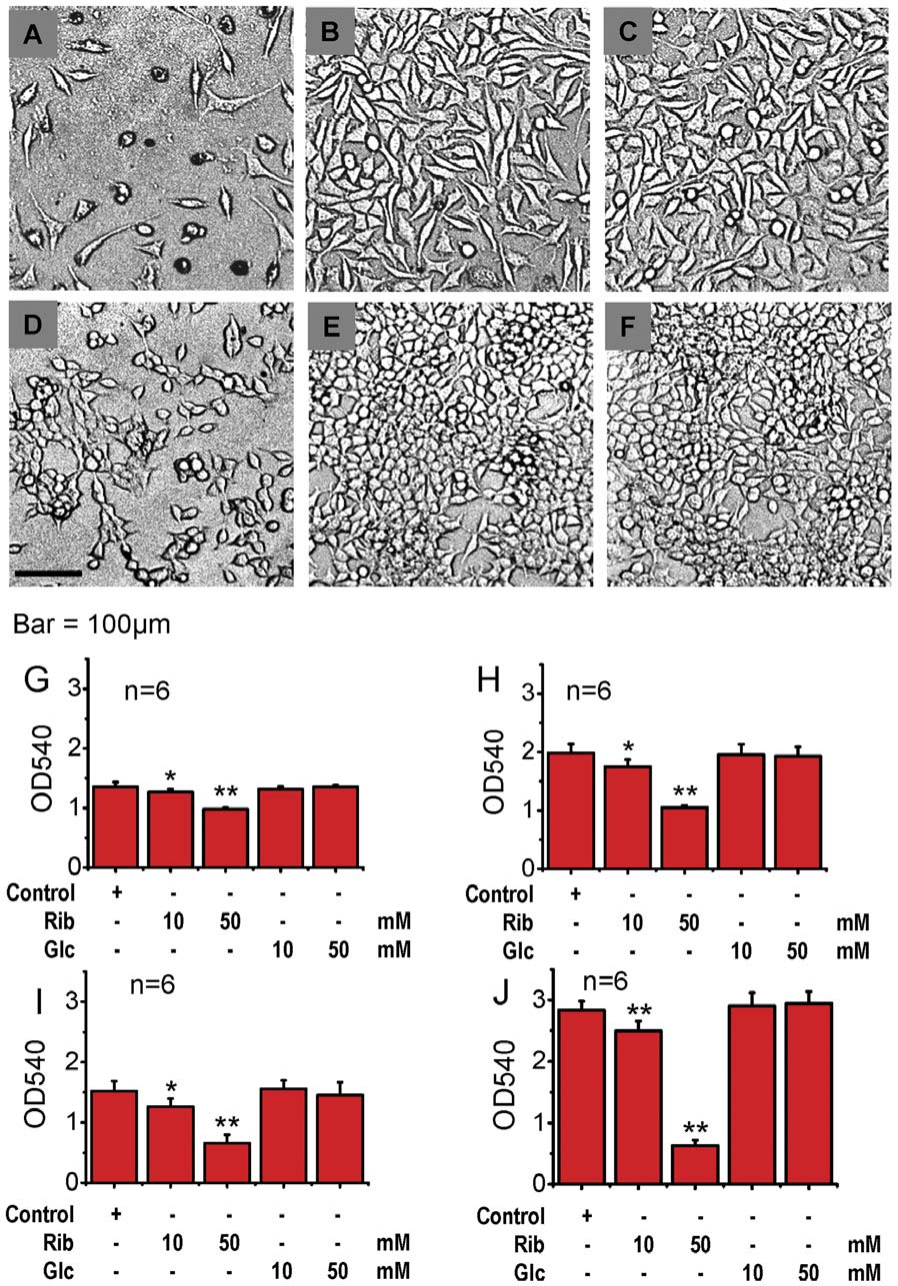
Changes in cell viability in the presence of D-ribose. The morphology of SH-SY5Y cells was observed by inverted contrast microscopy after incubation with 50 mM Rib (A), or 50 mM Glc (B) for 3 days. Untreated cells were used as controls (C). HEK293T cells treated with the same concentration of Rib (D), Glc (E) and control cells (F) were imaged under the same conditions. SH-SY5Y (G and H) and HEK293T (I and J) cells were incubated with Rib or Glc as indicated and cell viability was measured using the MTT assay at day 2 (G and I), and day 3 (H and J) after addition of the monosaccharides.

To investigate whether Rib leads to decreases in cell viability, SH-SY5Y human neuroblastoma (SH-SY5Y) cells and Human embryonic kidney 293T (HEK293T) cells were incubated with D-ribose or D-glucose at different concentrations. Cell viability was measured by MTT assays at 2 and 3 days after addition of the monosaccharide. After 2-day treatments, the viability of both cell lines decreased significantly in media containing 10 mM (P<0.05) or 50 mM (P<0.01) Rib, but it did not change markedly in the presence of Glc compared with the control (Fig 1G and I). MTT assays also gave the same results after 3 days of Rib treatment (Fig 1H and J). Furthermore, the number of SH-SY5Y and HEK293 cells was markedly lower after treatment with 50 mM Rib for 3 days compared with Glc-treated and control cells (Fig 1A to F).

**Figure 2:**
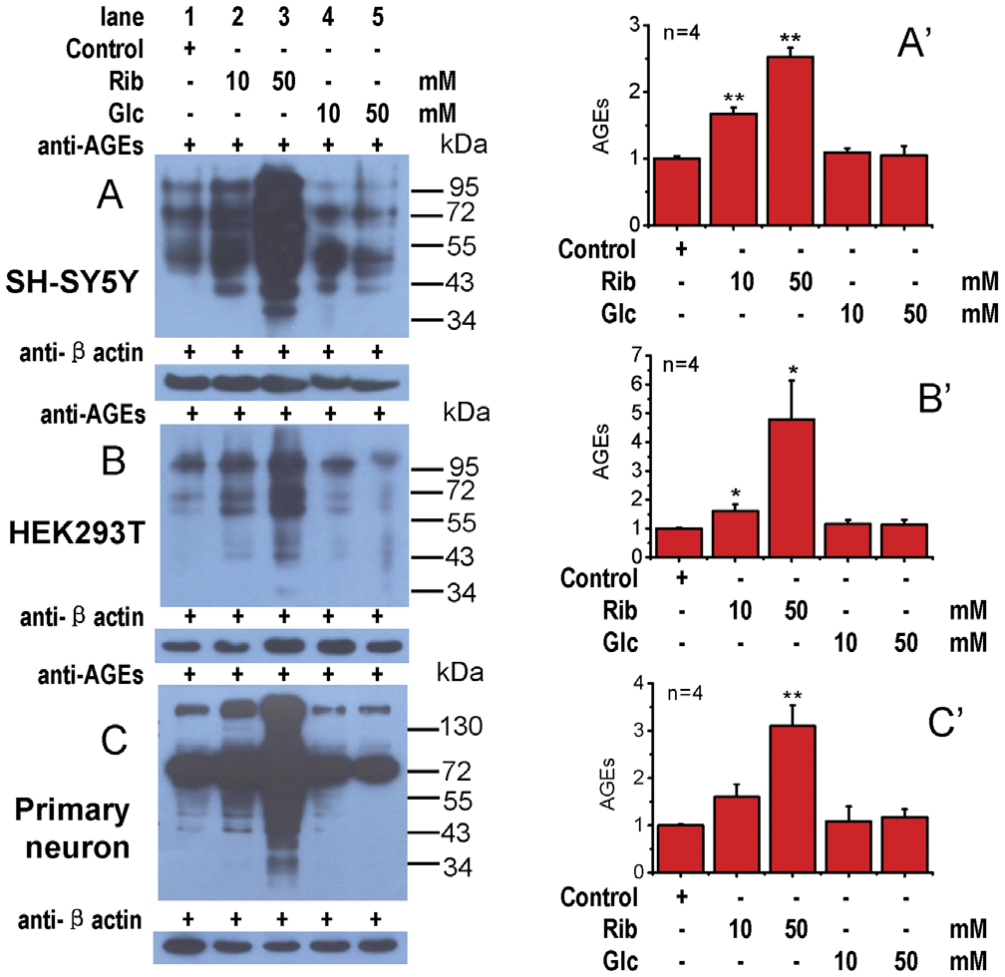
Elevation of AGEs in cells in the presence of D-ribose. SH-SY5Y cells (panel A), HEK293T cells (panel B) and primary cultured rat neurons (panel C) were treated with Rib and Glc as indicated for 2 days. AGEs were detected with anti-AGEs (6D12 monoclonal antibody). β-Actin was used as a loading control. Quantification results are shown in panels A′, B′ and C′ respectively. The control value was set as 1.0. All values are expressed as means ± S.E.M. * P<0.05, ** P<0.01.

It is known that AGEs have cytotoxicity [15], [17] and can inhibit cell proliferation [18]. Rib and Glc react with protein amino groups to initiate a non-enzymatic glycation process which results in AGE formation. Thus, we detected the presence of AGEs in SH-SY5Y, HEK293 cell lines and primary cultured hippocampal neurons incubated with Rib for 2 days by Western blotting. As shown in Fig 2A, the level of AGEs in SH-SY5Y cells was markedly (P<0.01) increased in the presence of Rib at concentrations of 10 mM or higher. However, in the presence of Glc, the level of AGEs did not increase significantly under the experimental conditions used. Similarly, the level of AGEs in both HEK293T cells and primary cultured hippocampal neurons was also enhanced significantly after Rib treatment (Fig 2B and C). These results indicate that Rib is much more active in protein glycation resulting in high yields of AGEs and reduced cell viability.

### Serum glycated proteins and AGEs significantly increase in D-ribose-treated mice

Having determined that Rib but not Glc is able to glycate proteins rapidly and produce high levels of AGEs in cultured cells *in vitro*, we investigated whether Rib is able to induce AGE formation *in vivo*.

**Figure 3:**
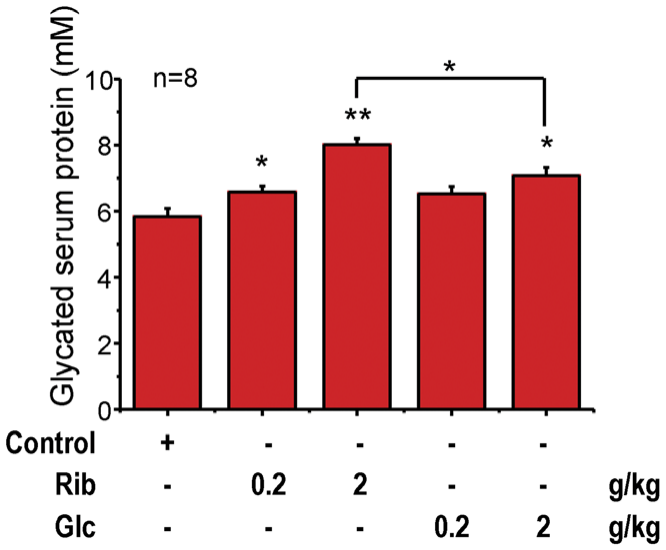
Intraperitoneal injection of D-ribose results in an increase in the concentration of glycated serum protein. Mice were injected (i.p.) with Rib as indicated for 30 days and serum was taken for assays of glycated serum protein. Mice injected with Glc and saline were used as controls. * P <0.05, ** P<0.01.

1. **D-ribose treatment does not cause significant dysfunction of the liver or kidneys.** To compare the ability of Rib and Glc to elevate the level of AGEs *in vivo*, we intraperitoneally injected C57BL/6J mice with D-ribose or D-glucose (0.2 or 2 g/kg sugar; control group injected with saline) for 30 days. None of the sugar treatment groups showed any significant visual abnormalities and they gained weight normally within the period of treatment (Table 1). There were no significant changes in serum ALT or AST in both Rib- and Glc-treated subjects (Table 2). Furthermore, serum creatinine concentrations did not markedly vary with the injections. These results indicate that treated mice did not suffer from liver and kidney damage under the experimental conditions used.
2. **Glycated serum proteins and AGEs increase after injection with D-ribose.** As shown in Table 1, blood glucose concentrations did not change markedly in Rib-treated mice though those of the two Glc-treated mice groups were elevated significantly (P<0.05). However, the amount of glycated serum protein was significantly increased in the blood of mice intraperitoneally injected with Rib at 0.2 g/kg (P<0.05) and 2g/kg (P<0.01) (Fig 3). Glycated serum proteins increased with 2 g/kg Glc injections (P<0.05), but not with low dose (0.2g/kg) Glc injections in the Glc-treated mice groups. Furthermore, the concentration of glycated serum proteins after Rib injections (2 g/kg) was significantly higher than that after Glc-treated injections (P<0.05) under the experimental conditions used. These results also suggest that Rib has a faster glycation rate than Glc *in vivo*.

**Table 1:** Body weight and serum glucose concentration.

Treatment	Body weight (pre-injection) (g)	Body weight (post-injection) (g)	Serum glucose concentration (mM)
**Control**	18.72±0.62	25.58±1.48	7.11±0.45
**0.2g/kg Rib**	18.35±0.34	25.43±1.41	7.51±0.35
**2g/kg Rib**	18.85±0.72	24.23±1.21	7.36±0.24
**0.2g/kg Glc**	18.67±0.87	25.03±1.57	7.85±0.27*
**2g/kg Glc**	18.76±0.95	25.00±1.55	8.04±0.61*

Mice were injected (i.p.) with Rib or Glc as indicated for 30 days. Body weight pre- and post-treatment is shown in grams. After treatment, serum was collected for assaying glucose concentration. All values are expressed as means ± S.E.M.

*P<0.05.

**Table 2:** Serum ALT and AST activity and serum creatinine concentration.

	Rib		Glc		Control
	0.2g/kg	2g/kg	0.2g/kg	2g/kg	
**ALT**	47.6±12.84	30.8±4.13	34.4±4.17	42±6.72	41.2±9.02
**AST**	48.1±7.26	38.1±9.36	52.2±5.11	48.8±3.06	53.7±8.47
**Cre**	41.5±1.28	42.6±0.28	40.8±1.43	41.6±0.41	41.9±0.51

After 30 days of treatment, serum was collected to assay the activity of ALT and AST and creatinine concentration. All values are expressed as means ± S.E.M.

**Figure 4:**
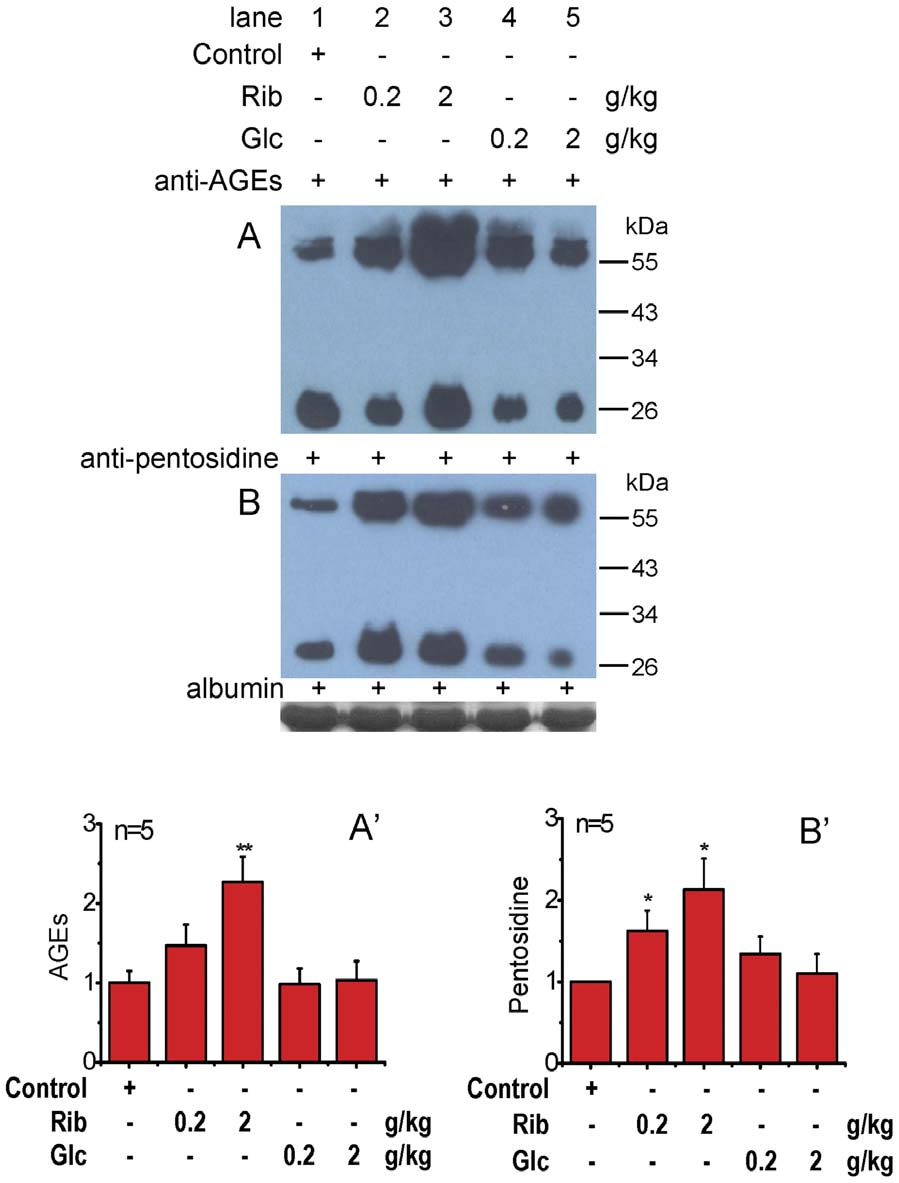
Changes in serum AGEs with intraperitoneal injection of D-ribose. Conditions for the injection of monosaccharides were the same as those given in Fig 3, except that serum AGEs were detected with an anti-AGEs monoclonal antibody (panel A) and an anti-pentosidine monoclonal antibody (panel B). Serum albumin level was used as a loading control. Quantification results are shown in panels A′ and B′, respectively. The saline control value was set as 1.0. All values are expressed as means ± S.E.M. * P<0.05, ** P<0.01.

Having established that injection of Rib leads to an increase in glycated serum proteins, we measured changes in serum AGE formation in mice treated with Rib and Glc to determine whether high levels of glycation lead to AGE production (Fig 4). Strikingly, serum AGEs were markedly elevated in the sera of mice that had been injected with Rib. Those treated with Glc were not significantly different from the control group. Similar results were also obtained when the anti-pentosidine antibody was used. Serum pentosidine level was markedly increased in the presence of Rib (both 0.2 g/kg and 2 g/kg) (P<0.05). These results demonstrate that Rib significantly elevates the glycation of proteins in the blood resulting in accelerated AGE formation under our experimental conditions.

### D-ribose-treated mice have a marked increase in brain AGEs

**Figure 5:**
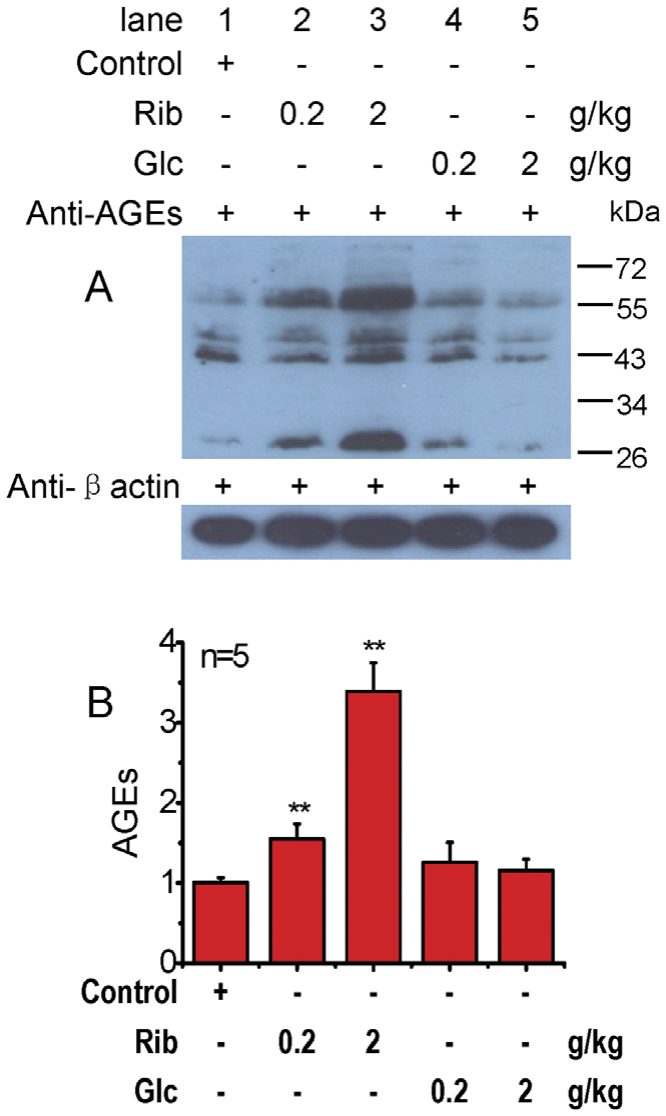
Enhancement of brain AGEs injected with D-ribose. Conditions for the injection of Rib were the same as those given in Fig 3, except that AGEs in the mouse brain were detected by Western blotting using anti-AGEs (6D12 monoclonal antibody, panel A). β-Actin level was used as a loading control. Quantification results are shown in panel B. The saline control value was set as 1.0. All values are expressed as means ± S.E.M. * P<0.05, ** P<0.01.

Rib can pass through the blood-brain barrier and enter the brain by simple diffusion [19]. To investigate whether injected Rib can elevate the glycation of proteins in the brain, we measured AGEs in the mouse brain by Western blotting. As shown in Fig 5, intraperitoneal injection of Rib led to the formation of significantly more AGEs in the mouse brain. However, Glc did not have a significant effect on AGE formation in the brain compared to the control. This suggests that Rib can react effectively with proteins and increase AGEs in the mouse brain.

**Figure 6:**
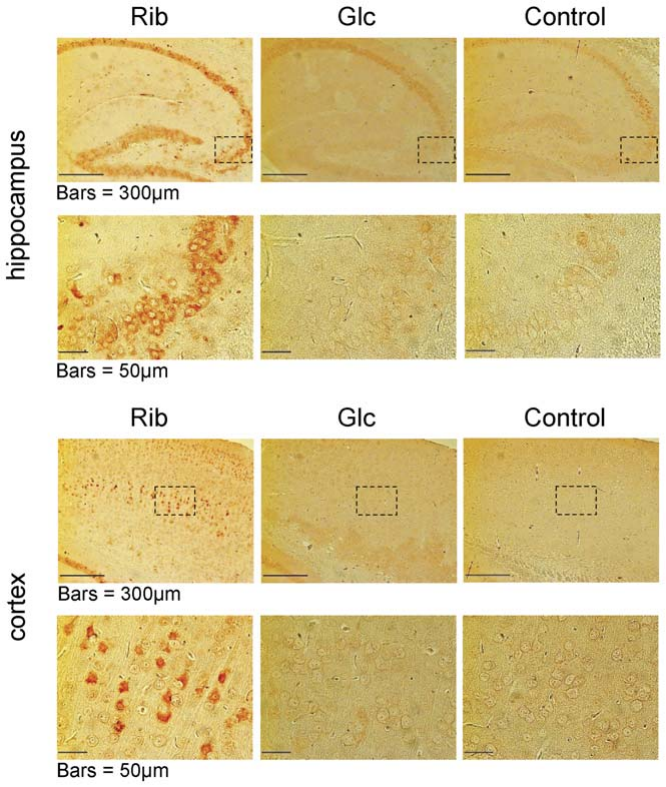
Immunohistochemical staining of AGEs in the hippocampus and cortex. Mice were injected (i.p.) with Rib at dose of 2 g/kg, or Glc at dose of 2 g/kg, or 0.9% saline (controls) for 30 days. AGEs in the mouse brain were detected by immunohistochemistry using anti-AGEs monoclonal antibody. The areas outlined with dashed lines are magnified in the lower panels (bar  =  50 µm).

To confirm the effect of Rib on accelerating the formation and accumulation of AGEs in the brain, we performed immunohistochemistry staining on microtome sections of the mouse brain (Fig 6). Compared with the control group, AGEs were observed to increase throughout the hippocampus of mice that had been injected with Rib for 30 days. However, no obvious differences in the hippocampus were found in the Glc-treated and control mice groups. Furthermore, AGE signals were more clearly evident in the cortex of Rib-treated mice, compared with those treated with Glc. This indicates that the rapid formation of Rib-induced AGEs occurred in both the hippocampus and cortex.

**Figure 7:**
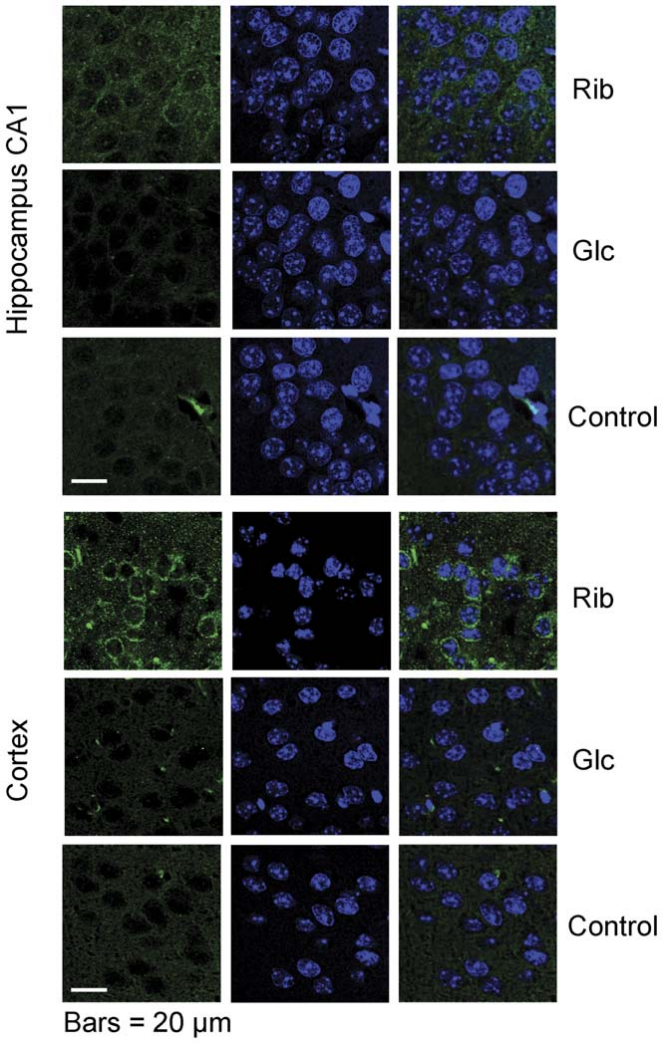
Immunofluorescent staining of AGEs in the hippocampus and cortex. Mice were injected (i.p.) with Rib at dose of 2 g/kg, or Glc at dose of 2 g/kg, or saline (controls) for 30 days. AGEs in the mouse brain were detected by immunofluorescent staining. The brain sections were double-labeled for AGEs (green) and nuclei (blue).

We used immunofluorescent staining to further demonstrate that Rib is able to induce AGE formation in the mouse brain. As shown in Fig 7, AGE signals were clearly visible in the cortex of mice treated with Rib but not in those treated with Glc or saline. The fluorescent signals of AGEs were mainly localized outside the nucleus. Similar results were also observed in the hippocampus of the Rib group though the signals were relatively lower than those in the cortex.

### Impairment of spatial learning and memory in the Morris water maze

**Figure 8:**
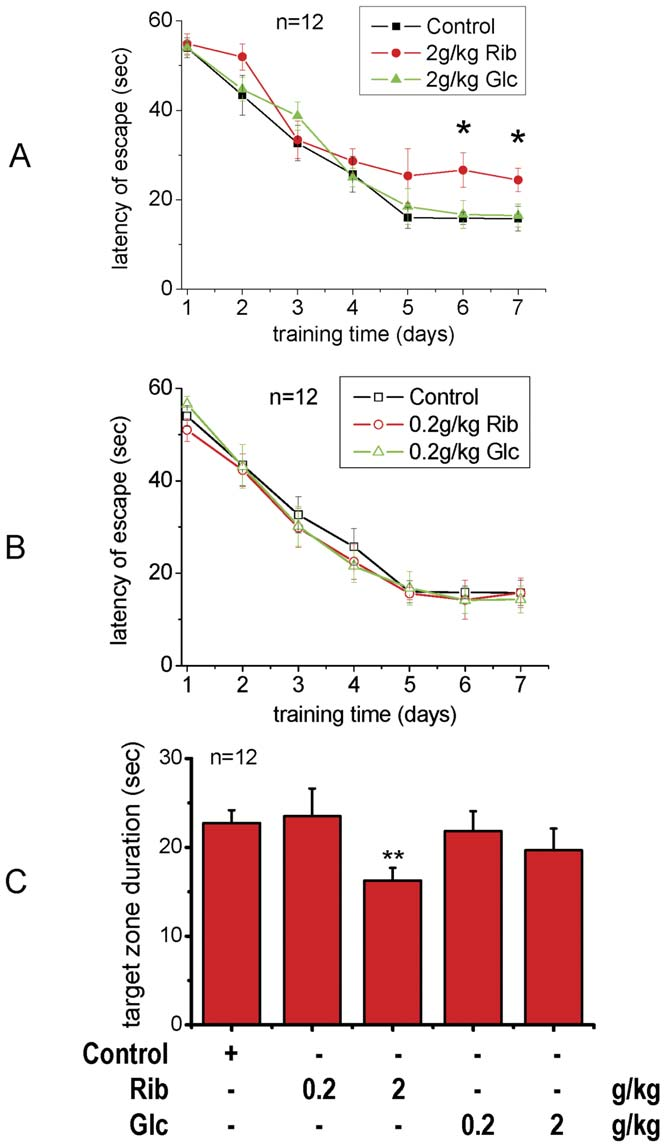
Decline in the performance of mice injected with D-ribose in the Morris water maze test. Conditions for the injection of Rib were the same as those given in Fig 3. The length of time mice took to find the hidden platform was recorded as latency of escape during each of the seven training days (panel A and B). The length of the searching time spent in the quadrant when the platform was removed during the probe trial is shown in panel C. All values are expressed as means ± S.E.M. * P<0.05, ** P<0.01.

AGEs, which have been found in the brains of senile dementia patients [20], are cytotoxic [12], [17]. To assess changes in the spatial learning and memory of mice whose brain AGE levels were elevated after injection of Rib, we tested their behavior in the Morris water maze. During the training session, all mice improved their performance as indicated by shortened escape latencies over successive days, and mice from each treatment group had the same level of performance (no significant individual effect was observed in the first three trials on day 1) prior to treatment. Escape latencies of mice injected with Rib (0.2 g/kg) were not significantly different compared with the control group (Fig 8B). However, the escape latency of mice injected with Rib (2 g/kg) on days 6 and 7 was higher than that of control mice (P<0.05), but there was no significant difference between the control group and the Glc (2 g/kg) -treated group (Fig 8A).

Withdrawal of the platform induced a general tendency to swim in the quadrant where the platform was previously located and in the platform zone, in preference to other equivalent zones. Control, Rib (0.2 g/kg) -treated and Glc (both 0.2 g/kg and 2 g/kg) -treated mice spent significantly more time swimming in the target quadrant (where the platform was located) (no significant differences were observed among these four groups), than Rib (2 g/kg) -treated mice (P<0.01), whose time in each quadrant was more evenly distributed (Fig 8C). These results indicate that spatial learning and memory ability in Rib-treated mice are significantly impaired.

## Discussion

As a reducing saccharide, Rib reacts with protein amino groups to initiate a post-translational modification process widely known as non-enzymatic glycation [1]. This reaction proceeds from reversible Schiff bases to stable, covalently-bonded Amadori rearrangement products. Once formed, the Amadori products undergo further chemical rearrangements to form irreversibly bound AGEs, which are a heterogeneous group of structures including pyrraline, pentosidine, crossline, and carboxymethyl-lysine [3]. As described previously, Rib is much more active in protein glycation than Glc *in vitro* [12], [15], [16]. Here, we also found that Rib reacts rapidly with proteins and showed that Rib treatment results in a significantly higher level of AGEs both in cultured cells and in the mouse serum and brain. This demonstrates that AGEs result from ribosylation not only in mixtures of Rib and proteins in a test-tube, but also in cultured cells, and in the mammalian serum and brain.

Even though glycation of proteins with reducing saccharides has been widely studied, the formation of monosaccharide-induced intracellular AGEs has not previously been observed. Here, 10 mM Rib enhanced AGE formation in cultured cells, and diminished cell viability. This work is the first to show that Rib enhances the yield of AGEs in HEK293T and SH-SY5Y cells and primary cultured hippocampal neurons. Rib showed significantly higher cytotoxicity than Glc in cell culture. The high cytotoxicity of Rib may result from the rapid formation of AGEs as a result of ribosylation under these experimental conditions.

Furthermore, this monosaccharide also enhances the yield of AGEs in both the hippocampus and cortex of the mouse brain after intraperitoneal injection. Impairment of spatial cognition was observed to be coincident with these increases in intracellular AGEs when Rib-treated mice were tested in the Morris water maze. Glc, however, was unable to elevate the yield of AGEs under our experimental conditions. This clearly demonstrates that an overload of Rib may result in a high level of AGEs in the brain and neurons. We would like to emphasize that Rib is much more effective than Glc in the glycation of proteins not only in vitro, but also in vivo. The intracellular AGE-enhanced cell model and the AGE-enhanced cognitive impairment mouse model successfully established here can be used for further investigation of the mechanisms behind the phenomena observed.

As shown in the results of Western blotting (Fig 2 and 5), a low level of glycated proteins or AGEs is already present under physiological conditions in both cells and the mouse brain since cells and blood contain certain concentrations of reducing saccharides. In normal human serum, approximately 6 to 10% of the albumin is glycated [21], while in hyperglycemia, this proportion typically increases two- to three- fold [22].

High levels of Rib are not only able to enhance AGE formation *in vivo*, but also induce dysfunction of spatial cognition. This is the first time that Rib-induced cognitive impairment has been observed in mice. The spatial learning and memory ability of mice markedly declined after 30 days of Rib administration. However, those that were injected with the same concentration of Glc did not show significant impairments of spatial cognition compared with saline controls. This suggests that Rib overload for a relatively long period may be harmful to brain function. This treatment paradigm of inducing high levels of AGEs in the brain by administration of Rib can be used as a model for the study of dementia.

Here, we believe that the decline in learning and the loss of memory are due to ribosylation and its resultant AGEs; glycation with Rib induces mouse spatial cognitive impairment. This viewpoint is based on the following observations and data. (1) Our previous work has shown that Rib is much more active in protein glycation than Glc *in vitro* [12], [15]. (2) Proteins become highly cytotoxic after they are ribosylated [12], [15]. (3) Rib decreases the cell viability and enhances the level of AGEs in HEK293, SH-SY5Y cells and primary cultured hippocampal neurons. (4) Rib-injected mice do not have severe liver and kidney damage, but do have impairment of cognitive function (Table 2). (5) As described previously, Rib can pass through the blood-brain barrier and react with cellular molecules [19]. (6) Rib elevates the level of AGEs in the mouse hippocampus and cortex. Finally, (7) Decline in learning and memory is observed in Rib-injected mice, but not in Glc-injected and control mice.

AGEs were clearly evident in the hippocampus and cortex of Rib-injected mice (Fig 6). Glc, however, did not significantly elevate AGEs in the mouse brain under the same conditions. Toxic AGEs have previously been implicated in diabetes [3], cataracts [23], renal failure [24], and other disorders [25]. Immunohistochemical studies have demonstrated the presence of AGEs in the senile plaques of brains from Alzheimer' disease patients [20], suggesting a link between AGEs and senile plaque formation. Takeda and colleagues have reported that AGE deposits are markedly increased in the hippocampus and the parahippocampus of the brains of Alzheimer's disease patients [26]. Furthermore, it has recently become clear that glycation is also involved in other neurodegenerative diseases and cognitive disorders [27]. This indicates that an AGE overload in the brain may be related to the dysfunction of learning and memory.

The relationship between AGE accumulation, cognitive decline and neurodegenerative disease is still under active investigation. Multiple studies have suggested that AGEs are directly neurotoxic to cultured neurons [15], [17]. AGEs and their precursors (methylglyoxal and glyoxal) may increase the aggregation and cytotoxicity of intracellular amyloid-beta carboxy-terminal fragments [28]. AGEs, as a kind of specific ligand, can also interact with receptors for advanced glycation end products (RAGE) and activate an array of signal transduction cascades [29]. By the interaction with RAGE, AGEs may be involved in the generation of ROS and inflammation, and may play a role as activating factors for neuroglia cells such as astrocytes or microglias [30], [31], inducing them to produce cytotoxic cytokinelike molecules which then may induce further neuronal cell injury and death and dysfunction of the brain. However, whether AGEs induced by Rib are the same as those generated spontaneously *in vivo*, and the mechanism by which Rib-induced AGEs impair spatial learning and memory, need further investigations.

As a readily available source of energy, ribose is used to improve athletic performance and the ability to exercise by boosting muscle energy. It has also been used to improve symptoms of conditions such as chronic fatigue syndrome, fibromyalgia, and coronary artery disease [32], [33], [34]. Rib, as a bioactive ingredient is used widely in nutrition and medicine. Large quantities of Rib are consumed as health supplements and in functional food and beverage formulations each year. However, Rib is very active in the glycation of proteins and its associated chronic risks should be taken into consideration. Our results have shown that administration of high doses of Rib over a long period can lead to high yields of AGEs *in vivo* and cognitive dysfunction. Glycation affects the biological functions of proteins and crosslinking by glycation results in the formation of detergent-insoluble and protease-resistant aggregates. Thus, the effects of ribosylation on the human brain should be regularly examined during long term administrations of Rib as a drug. Our findings on the importance of ribosylation are relevant to medical professionals monitoring the therapeutic use of Rib.

In summary, we have shown that Rib rapidly reacts with proteins and produces AGEs in cells, inducing a decrease in cell viability. Administration of Rib to mice leads to the accumulation of a significantly high concentration of AGEs in the brain and subsequent impairment of spatial learning and memory ability. Administration of Rib can thus be used to establish a mouse model of dysfunction in spatial cognition.

## Materials and Methods

### Ethics Statement

The handling of mice and experimental procedures were approved by the Animal Welfare and Research Ethics Committee of the Institute of Biophysics, Chinese Academy of Sciences (Permit Number: SYXK2010-128).

### Cell culture and treatment

Human embryonic kidney 293T (HEK293T) cells [35] and SH-SY5Y human neuroblastoma (SH-SY5Y) cells [36] were obtained from Cell Resource Center (IBMS, CAMS/PUMC, China). HEK293T and SH-SY5Y cells were cultured in Dulbecco's modified Eagle' medium (Gibco, USA) supplemented with 100 IU/ml penicillin and 100 µg/ml streptomycin at 37°C in a humidified 5% CO_2_ incubator as described [37], [38]. The medium contained 10% fetal bovine serum (Gibco, USA). Cells were grown to 70–80% confluence in 25 mm diameter dishes. Primary hippocampal neuron cultures were prepared from 18-day-old Sprague-Dawley rat embryos as described previously [39]. Mature hippocampal neurons (cultured for 14 days) were prepared for treatment. For all experiments, the culture medium was replaced with serum-free medium before Rib or Glc treatment. Cells were incubated with Rib (Amresco, USA) or Glc (Sigma, USA) at concentrations of 10 mM and 50 mM for 48 hours. Cells were then collected to prepare cellular extracts for Western blots.

### Cell viability test

To determine cell viability, we used the standard 3-(4, 5-dimethylthiazol-2-yl)-2, 5-diphenyl tetrazolium bromide (MTT; Sigma, USA) test, with the slight modifications suggested by Mayo and Stein [40]. HEK293T or SH-SY5Y cells were seeded on 96-well plates at a concentration of ∼3000 cells per well. After 24 hours, the culture medium was replaced with serum-free medium in the presence of Rib or Glc at different concentrations. Medium without monosaccharides was used as a control. After 48 or 96 hours of treatment, MTT (final concentration 0.5 mg/ml) was added and the plates were incubated at 37°C for 4 hours. The reaction was stopped by replacement of the MTT-containing medium with 150 µl DMSO (Amresco, USA), and absorbance at 540 nm was measured on a Multiscan MK3 spectrophotometer (Thermo, USA).

### Animals and administration

Male C57BL/6J mice (8–10 weeks) were obtained from Vital River Laboratory Animal Technology Co. Ltd. (China). After 1 week of acclimatization to the cages, mice were randomly divided into five groups and received intraperitoneal injections each day for 30 days with Rib at doses of 0.2 or 2 g/kg, or Glc at doses of 0.2 or 2 g/kg, or 0.9% saline (controls). All mice were maintained in animal facilities under pathogen-free conditions.

### Morris water maze test

After 30-days of injections, the Morris water maze (MWM) test was performed as described previously [41]. The experimental apparatus consisted of a circular water tank (90 cm in diameter, 35 cm in height), containing water (23±1°C) to a depth of 15.5 cm, which was rendered opaque by adding ink. A platform (4.5 cm in diameter, 14.5 cm in height) was submerged 1 cm below the water surface and placed at the midpoint of one quadrant. The water tank was located in a test room, which contained various prominent visual cues. Each mouse received three periods of training per day for seven consecutive days. Latency to escape from the water maze (finding the submerged escape platform) was calculated for each trial. On day 8, the probe test was carried out by removing the platform and allowing each mouse to swim freely for 60 seconds. The time that mice spent swimming in the target quadrant (where the platform had been located during hidden platform training) was measured. All data were recorded with a computerized video system.

### Sample collection

After behavioral testing, mice were sacrificed and their blood (about 0.7 ml per mouse) was collected as described previously [42] and centrifuged (2000×g, 20 minutes, 4°C). Serum was aspirated and stored at −70°C until assayed, as described below. At the same time, the brain was quickly dissected out, immediately homogenized in lysis buffer (Beyotime, China) and then centrifuged to yield supernatants for Western blots, or fixed in 4% paraformaldehyde for immunohistochemistry experiments.

### Serum physiochemical assays

Glycated serum protein (GSP) [43] and blood glucose [44] were measured using kits obtained from the Nanjing Jiancheng Bioengineering Institute (China) according to the manufacturer' guidelines. The activity of the serum enzymes alanine aminotransferase (ALT) [45], aspartate aminotransferase (AST) [46] and serum creatinine [47] was determined using a spectrophotometric diagnostic kit from Biosino Biotechnology Company Ltd. (China).

### Gel electrophoresis and Western blotting

The level of AGEs or pentosidine was determined in cultured cells, brain tissues, and mice sera as described previously [12]. Sample protein concentrations were quantified with the BCA™ Protein Assay Kit (Pierce, USA). Equivalent amounts of protein (20–30 µg) were resolved on 12% SDS-PAGE gels and transferred to nitrocellulose membranes. Membranes were then incubated respectively with anti-AGE 6D12 monoclonal antibody (TransGenic, Japan), anti-pentosidine PEN12 monoclonal antibody (TransGenic, Japan) or anti-β actin monoclonal antibody (Sigma, USA) overnight at 4°C. Each membrane was washed three times with PBS with 0.1% (v/v) Tween-20 (PBST, pH 7.4), then incubated with horseradish peroxidase-conjugated anti-mouse IgG for 1 hour at 37°C. The membranes were again washed three times with PBST, and then immunoreactive bands were visualized using enhanced chemiluminescence detection reagents (Applygen, China). The protein bands were visualized after exposure of the membranes to Kodak X-ray film and quantified by Quantity One 1D analysis software 4.5.2 (Bio-Rad, USA).

### Immunohistochemistry and immunofluorescent staining

As described [31], mice brains were immersed in 4% paraformaldehyde for 48 hours immediately after they were dissected out. After fixation, brains were embedded in paraffin blocks. Five to eight micrometer thick sections were processed for immunohistochemical analyses. Deparaffinized and hydrated sections were incubated in Target Retrieval Solution at 95°C for 30 minutes for enhancement of immunoreactivity, then permeabilized with 0.3% H_2_O_2_ in absolute methanol for 10 minutes to block endogenous peroxidase, and incubated in 10% normal goat serum in PBS at room temperature for 30 minutes. The specimens were incubated overnight at 4°C in anti-AGEs 6D12 monoclonal antibody solution diluted in PBS. After washing with PBS, sections were subsequently incubated with biotin-labeled secondary antibodies (37°C, 1 hour). The immunoreaction was detected using horseradish peroxidase-labeled antibodies (37°C, 1 hour) and red staining was visualized with an AEC system (Nikon Optical, Japan).

Immunofluorescent staining was performed as described [48]. After deparafinization, hydrating and immunoreactivity enhancement, sections were incubated in 10% normal goat serum in PBS at room temperature for 30 minutes and probed overnight at 4°C with anti-AGE 6D12 monoclonal antibody diluted in PBS. Bound antibodies were visualized with Alexa 488-conjugated anti-mouse IgG (Invitrogen, USA) and cell nuclei were stained with the DNA-specific fluorescent reagent Hoechst 33258. Immunolabeled tissues were observed under an Olympus FV500 laser scanning confocal microscope (Olympus, Japan).

### Data analysis

All values reported are means ± standard errors (SE) unless otherwise indicated. Data analysis was performed by one way analysis of variance (ANOVA) using Origin 7.0 statistical software. Differences with a probability level of 95% (P<0.05) were considered significant.
